# Molecular basis of HFE-hemochromatosis

**DOI:** 10.3389/fphar.2014.00042

**Published:** 2014-03-11

**Authors:** Maja Vujić

**Affiliations:** Institute of General Zoology and Endocrinology, University of UlmUlm, Germany

**Keywords:** Hfe, HH, hepcidin, extra-hepatic Hfe, Bmp/Smad signaling

## Abstract

Iron-overload disorders owing to genetic misregulation of iron acquisition are referred to as hereditary hemochromatosis (HH). The most prevalent genetic iron overload disorder in Caucasians is caused by mutations in the *HFE* gene, an atypical MHC class I molecule. Recent studies classified HFE/Hfe-HH as a liver disease with the primarily failure in the production of the liver iron hormone hepcidin in hepatocytes. Inadequate hepcidin expression signals for excessive iron absorption from the diet and iron deposition in tissues causing multiple organ damage and failure. This review focuses on the molecular actions of the HFE/Hfe and hepcidin in maintaining systemic iron homeostasis and approaches undertaken so far to combat iron overload in HFE/Hfe-HH. In the light of the recent investigations, novel roles of extra-hepatocytic *Hfe* are discussed raising a question to the relevance of the multipurpose functions of *Hfe* for the understanding of HH-associated pathologies.

## AN ADEQUATE SUPPLY OF IRON IS A PREREQUISITE FOR GOOD HEALTH

Iron overload disorders comprise a wide range of inherited and acquired disorders of iron metabolism. Hereditary hemochromatosis (HH) encompasses a heterogenous group of inherited iron overload disorders with distinct underlying molecular defects and varying clinical symptoms (Camaschella and Poggiali, 2011). HH begins as mere iron overload which over time can cause serious organ dysfunctions leading to liver failure and cirrhosis, hepatocellular carcinoma, atherosclerosis, arthritis, fatigue, various endocrinopathies including diabetes, heart problems (both arrhythmia and cardiomyopathy, or loss of cardiac muscle function), hypermelanotic pigmentation of the skin, or compromised immune defense (Davies and Enns, 2004; Camaschella, 2005; Guggenbuhl et al., 2005; Pietrangelo, 2010). If remained untreated, HH is a life-threatening disorder. The current mainstay therapy for HH is phlebotomy (venesections), a relatively simple and inexpensive treatment, whereby blood is removed on a weekly basis for several months or more depending on the iron levels. For patients undergoing phlebotomy, liver, and cardiac functions improve after iron-depletion, whereas other HH-associated pathologies (e.g., diabetes mellitus or arthropathy) are often unchanged by the treatment (Brissot and de Bels, 2006; Sahinbegovic et al., 2010). In certain cases, iron chelation therapy may be taken into consideration (Brissot and de Bels, 2006). All current therapies for HH focus exclusively at manipulating the excess of iron.

## LOW LEVELS OF HORMONE HEPCIDIN HALLMARK HH

Genetic data in mice and patients demonstrated that a relative deficiency of hormone hepcidin underlies HH disorders (Ahmad et al., 2002; Bridle et al., 2003; Muckenthaler et al., 2003; Niederkofler et al., 2005; Wallace et al., 2005; Babitt et al., 2006; Lesbordes-Brion et al., 2006; Wallace et al., 2009). Hepcidin is a liver-derived peptide that oversees systemic iron changes by maintaining plasma iron levels within a narrow physiological range. It does so by determining iron absorption and iron release from the cells through binding to its receptor, the iron-export protein ferroportin (Nemeth et al., 2004; Nemeth and Ganz, 2006). Low levels of hepcidin signal for increased ferroportin activity thereby enhancing iron uptake from the diet via enterocytes, iron release from macrophages into the circulation and deposition of the excess of iron in parenchymal cells of tissues leading to a condition of systemic iron overload. The hepcidin-mediated ferroportin regulation can therefore be considered as the critical step for balancing systemic iron levels. Any disturbances in hepcidin regulators such as mutations in HH-genes like *HFE*, *Transferrin receptor 2 (TfR2*; Roetto et al., 2002; Kawabata et al., 2005; Nemeth et al., 2005),* Hemojuvelin (HJV*; Lanzara et al., 2004; Papanikolaou et al., 2004; Huang et al., 2005; Niederkofler et al., 2005; Babitt et al., 2006) or *Hepcidin *itself (Roetto et al., 2003; Lesbordes-Brion et al., 2006), and/or ferroportin (Pietrangelo, 2004; Sham et al., 2009; Le Lan et al., 2011) contribute to iron-related pathophysiology (Ganz and Nemeth, 2006).

## HFE-HH IS THE MOST COMMON TYPE OF HH IN WESTERN POPULATION

The most common type of hereditary HH is associated with the mutations in the *HFE *(High Fe) gene (Feder et al., 1996; Levy et al., 1999; Bridle et al., 2003; Muckenthaler et al., 2003). This is the most prevalent mutation in Western populations affecting approx. 1:250 individuals (Pietrangelo, 2010). The *HFE* gene was first identified in 1996 as a major histocompatibility (MHC) class I-like gene in which homozygosity for a missense mutation that results in cystein-to-tyrosine substitution at amino acid 282 of human HFE protein (C282Y), was found in vast majority of HH patients (Feder et al., 1996). Approximately 80% of HH patients are homozygous for C282Y and the frequency of this mutation decreases from the northwest to southwest Europe paralleling the settlements of ancient Celts (Distante et al., 2004). It is likely that mutation provided survival advantage against from what was then a very poor iron diet. Significantly fewer patients with a clinical diagnosis of HH are heterozygotes for a compound C282Y and H63D mutation (the latter is a substitution of aspartic acid for histidine at the position 63 of the HFE protein), whereas the homozygosity for H63D mutation usually causes little increase in iron absorption and rarely leads to the development of HH. Other mutations (e.g., S65C, V53M, V59M, H63H, Q127H, Q283P, P168X, E168Q, E168X, W168X) are rare and/or have low penetrance (Qaseem et al., 2005; Brissot et al., 2011).

The discovery of the *HFE* gene and the identification of the mutations associated with the HFE-HH were spectacular. The wealth of scientific and clinical data led towards more accurate diagnosis of HFE-HH, improved family screening and provided the first insights into the regulation of iron homeostasis by the *HFE*.

## HFE-HH IS THE LIVER DISEASE

The protein encoded by the *HFE* gene is a non-classical MHC class I-like protein which contains a signal sequence, peptide-binding extracellular domains, a transmembrane region, and a small cytoplasmic portion. Within the alpha-2 and alpha-3 extracellular domains are the four cysteine residues that form disulfide bridges representing one of the most conserved structural features of MHC class I molecules. HFE interacts with beta2-microglobulin and this association enables efficient transport of HFE to the cell surface where it interacts with TfR1. C282Y mutation disrupts the disulfide bridges in the extracellular domains of the HFE protein thereby preventing the association of HFE with beta2-microglobulin and TfR1. The lack of HFE interaction with TfR1 increases the affinity of TfR1 for the transferrin-bound iron thereby modulating iron absorption. In contrast to the C282Y mutation, mutant H63D HFE formed stable complexes with TfR1 being in line with the clinical data that H63D HFE mutations marginally affect iron absorption and rarely lead to HH.

The molecular link between the HFE protein and the TfR1 raised the possibility that this regulatory mechanism of iron transport may play a role in the pathogenesis of HH (Fleming et al., 1999; Anderson et al., 2002). The answer to whether Hfe alters cellular iron uptake by serving as a sensor mechanism in duodenal enterocytes was provided through the generation of mice bearing a selective deficiency of *Hfe *in the duodenal enterocytes (Vujic Spasic et al., 2007). Surprisingly, mice lacking *Hfe* in intestinal cells showed no iron accumulation in the liver nor hepatic hepcidin deficiency overruling the traditional hypothesis that duodenal Hfe played a gatekeeper role in controlling systemic iron homeostasis (Vujic Spasic et al., 2007). The question where *Hfe* acts to control iron homeostasis was revealed through the generation of mice with selective deficiency of *Hfe* in hepatocytes which recapitulated most of the anomalies within iron metabolism observed in constitutive *Hfe *mutant mice and HFE-HH patients (Adams, 2003; Wigg et al., 2003; Vujic Spasic et al., 2008; Adams and Barton, 2010). Mice deficient for the hepatocytic-*Hfe* show decreased hepcidin expression which led to uncontrolled iron uptake and iron accumulation in the liver (Vujic Spasic et al., 2008). Vice versa, hepatocellular transgenic over-expression of *Hfe* in mice lacking endogenous *Hfe* resulted in significant upregulation of hepcidin and normalization of transferrin saturation and liver iron levels (Schmidt et al., 2008). Collectively, these data establish hepatocytic-*Hfe* as the regulator of hepatic hepcidin expression and imply that regulatory cues involved in maintaining iron homeostasis are centered in the liver.

## HFE IS INVOLVED IN IRON-MEDIATED CONTROL OF HEPCIDIN EXPRESSION VIA THE BMP/SMAD SIGNALING PATHWAY

The above investigations argue for the role of Hfe in hepatocytes to regulate hepcidin expression and thus iron homeostasis. In the last couple of years first insights into to molecular mechanism coupling Hfe to hepcidin expression have begun to emerge.

**FIGURE 1:**
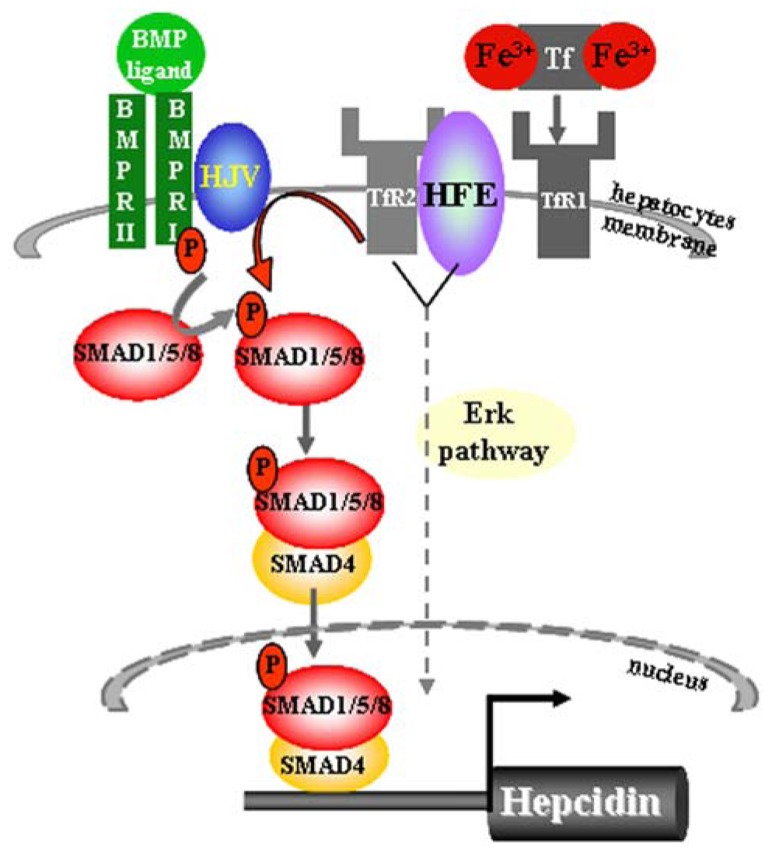
**Sensing of transferrin-bound iron and regulation of hepcidin expression in hepatocytes.** The iron-sensing process involves binding of transferrin-bound iron to TfR1 causing a dissociation of Hfe from the Hfe/TfR1 partnership, relocation of Hfe to TfR2 and presumably the formation of a large membrane-bound complex composed of Hfe/TfR2/Hjv and BMPRII and I. This hepatocyte-membrane complex activates transduction cascade involving the phosphorylation of the Smad1/5/8 and subsequent binding of common Smad4 protein to form a transcriptional complex which directly activates hepcidin transcription. The Bmp/Smad signaling is the central pathway for the regulation of hepcidin transcription. Lack of *Hfe* and other components of the membrane-bound complex severely impair the phosphorylation of Smad1/5/8 and consequently the transcription of hepcidin. Combined deficiency of *Hfe* and *TfR2* results in decreased Erk/Mapk signaling activity in the liver, implicating that additional or parallel signaling pathway to Bmp/Smad may be involved in the control of hepatic hepcidin transcription.

It is proposed that Hfe is sequestered by TfR1 protein in an iron-sensing complex located in the hepatocyte cell membrane (**Figure 1**) (Goswami and Andrews, 2006). The close interaction between Hfe and TfR1 is disrupted upon binding of circulating transferrin bound iron which binds with higher affinity to TfR1 (**Figure 1**) (Goswami and Andrews, 2006). *In vitro* studies proposed a model in which Hfe upon dissociation from Hfe/TfR1 complex, interacts with another HH protein, TfR2, and that this partnership is enlarged by the contribution of a membrane-bound bone morphogenetic protein (Bmp) co-receptor Hjv (**Figure 1**) (Goswami and Andrews, 2006; D’Alessio et al., 2012). However a direct interaction between Hfe and TfR2 could not be confirmed *in vivo* suggesting that TfR2 may regulate hepcidin expression in an Hfe-independent manner (Schmidt and Fleming, 2012). Importantly, Hfe-mediated hepcidin expression is abolished by the loss of endogenous Hjv protein implicating for co-dependency between Hfe and the Hjv (Schmidt et al., 2010). The Bmp co-receptor, Hjv, is involved in transmitting the signals initiated upon binding of Bmp ligands, the members of the multifunctional Tgf-β family proteins, to two cognate serine/threonine kinase receptors, type I and II located at the cell surface (**Figure 1**) (Miyazono et al., 2005; Babitt et al., 2006; Babitt et al., 2007). This interaction induces phosphorylation of the intracellular receptor-activated Smad proteins 1/5/8 and subsequent binding of common Smad4 protein to form an active transcriptional complex which directly regulates the expression of numerous target genes including hepcidin (**Figure 1**) (Miyazono et al., 2005; Babitt et al., 2006; Babitt et al., 2007; Truksa et al., 2009). The lack of *Hjv*, *Hfe*, *TfR2*, or combined *Hfe* and *TfR2* deficiency significantly impairs the activity of the Bmp/Smad-signaling pathway resulting in a low hepcidin expression and profound systemic iron overload (Babitt et al., 2006; Corradini et al., 2009, 2011; Kautz et al., 2009; Ryan et al., 2010; Vujic Spasic et al., 2013). Furthermore, mice deficient for hepatic Bmp type I receptors, *Alk2* or *Alk3* (Steinbicker et al., 2011), or *Smad4* expression (Wang et al., 2005), or for ubiquitous *Bmp6* expression (Andriopoulos et al., 2009; Meynard et al., 2009) present with relatively low hepcidin levels in regard to overall body iron overload. The Bmp/Smad signaling pathway therefore emerges as the central signaling event for regulating hepcidin transcription in hepatocytes. Recently, a contribution of the Erk/Mapk signaling cascade to hepcidin regulation has been proposed (Wallace et al., 2009) as its decreased signaling activity in the liver was associated with the combined deficiency of *Hfe* and *TfR2* suggesting that additional, or parallel signaling pathway to Bmp/Smad may control hepatic hepcidin transcription (**Figure 1**).

## THERAPEUTIC IMPLICATION OF ATTENUATED BMP/SMAD-MEDIATED HEPCIDIN REGULATION IN HFE-HH

Given the fact that hepcidin levels are low in Hfe-HH and that Hfe is involved in optimizing iron-response via Bmp/Smad signaling activity in the liver, it would be expected that by modulating hepcidin levels and/or the activity of the Bmp/Smad pathway, the iron homeostatic parameters in HFE-HH patients may be normalized. In contrast to phlebotomy therapies that manipulate the excess of iron, few pilot approaches were conducted with the aim to target the defective molecular mechanisms underlying the HFE-HH.

Short- and long-term hepcidin injections, or hepatic over-expression of hepcidin transgene in *Hfe*-deficient mice resulted in successful reconstitution of hepcidin expression to the levels present in wild type mice. Furthermore, high plasma iron levels present in *Hfe*^-^^/^^-^ mice were significantly reduced by hepcidin treatments, without affecting hepatic iron load which remained inappropriately high in regard to hepcidin levels (Nicolas et al., 2003; Laftah et al., 2004; Rivera et al., 2005; Viatte et al., 2006; Moran-Jimenez et al., 2010). Neither has exogenous Bmp6 administration to *Hfe*^-^^/^^-^ mice succeeded to reduce hepatic iron burden, although hepcidin expression was restored to the levels present in wild type mice, followed by a significant drop in serum iron levels and redistribution of iron in the spleen and duodenum in *Hfe*^-^^/^^-^ mice (Corradini et al., 2010). Due to severe side-effect (peritoneal calcification) that accompanied prolonged exogenous Bmp6 treatment in mice (Corradini et al., 2010), the Bmp6 substitution cannot be currently considered as an optional therapy for HH.

## HFE-HEPCIDIN-DEPENDENT AND -INDEPENDENT CONTROL OF IRON HOMEOSTASIS

**FIGURE 2:**
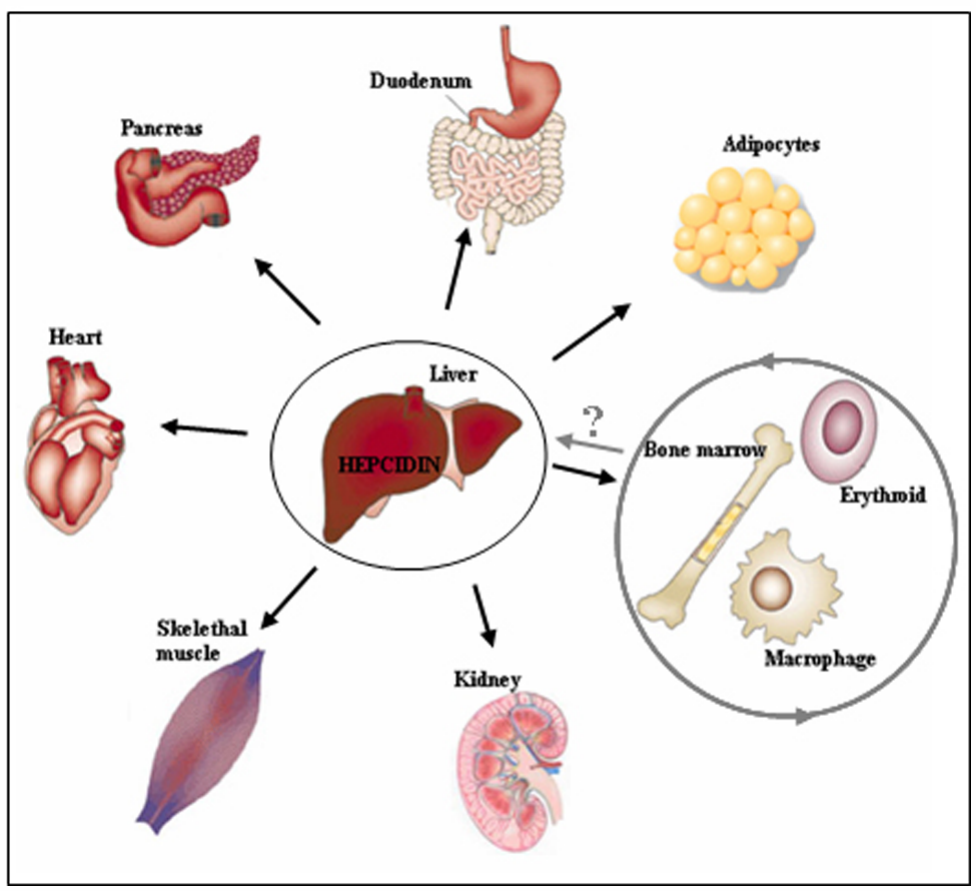
**Beyond the hepatocytes.** The regulatory cues controlling iron metabolism are centered in the liver where hepatocytic-*Hfe* directs the production of the liver iron hormone hepcidin. The lack of hepatocytic-*Hfe* leads to inadequate production of hepcidin which results in increased iron uptake by the duodenum, iron release from macrophages into the circulation and deposition of the excess of iron in numerous tissues causing systemic iron overload (indicated by black arrows). The actions of the *Hfe* in extra-hepatocytic cells, such as erythroid and macrophages (indicated by grey circle), have recently been proposed suggesting for previously neglected functions of the *Hfe* in these cells. These selective extra-hepatocytic functions of Hfe are involved in the control of local, tissue-specific iron homeostasis however their impact on systemic iron regulation and the relevance for the Hfe-HH associated pathologies remains still to be discovered.

The above data raise a critical question to whether Hfe-HH is an exclusive consequence of defective Bmp/Smad signaling and low hepcidin expression, or whether HH-associated pathologies may be intensified or refractory to iron-depletion strategies or hepcidin substitution if Hfe exerts distinct, *extra-hepatocytic functions* (**Figure 2**). Noteworthy, both Hfe and hepcidin are expressed in several extra-hepatic tissues (e.g., heart, skeletal muscle, kidney, lungs, thymus, duodenum) but so far, little is known whether Hfe-hepcidin regulation is operating in these cells and how it may impact on cellular and/or systemic iron homeostasis (**Figure 2**). For example, general *Hfe* deficiency was associated with better tolerance of *Hfe*^-^^/^^-^ mice to severe blood loss in regard to wild type mice or animals kept on an iron-rich diet (Ramos et al., 2011). During erythropoietic stress conditions hepcidin expression is severely hampered which in turn signals for enhanced iron absorption, iron mobilization from the stores and iron utilization by the erythron. It was proposed that in addition to general *Hfe* and hepcidin deficiency, selective *Hfe* actions in erythroid cell may contribute to overall better tolerance of *Hfe*^-^^/^^-^ mice to severe blood loss by enhancing transferrin-bound iron uptake and thus modulating erythroid iron homeostasis (Ramos et al., 2011) (**Figure 2**). However a direct role of *Hfe* in erythroid cells and its contribution to cellular and overall iron homeostasis remains still to be confirmed.

Furthermore, Hfe may be involved in controlling iron homeostasis in a *non-hepcidin dependent manner*. It was proposed that Hfe may act to control iron release or iron uptake in cells since expression of HFE protein in HT29 human colon carcinoma cells, THP-1 cells, or in monocytes derived from HFE-HH patients inhibited iron release from the cells and resulted in increased iron accumulation without affecting iron uptake (Montosi et al., 2000; Drakesmith et al., 2002; Davies and Enns, 2004). On the other hand, over-expression of HFE in HeLa and hepatic HepG2 cells decreased iron loading in these cells (Gao et al., 2008), suggesting that HFE may exert distinct iron-regulatory functions depending on the site of its expression. In line with this, recent *in vivo* studies demonstrated that *Hfe* actions in macrophages are not required for the control of hepatic hepcidin expression (Makui et al., 2005; Vujic Spasic et al., 2008) and that *Hfe* may display hepcidin-independent iron-modifying effect in these cells (Garuti et al., 2011) (**Figure 2**). Transplantation of *Hfe*^+^^/^^+^ donor liver into *Hfe*^-^^/^^-^ recipient mice improved hepatic hepcidin expression and reduced the liver iron burden in *Hfe*^-^^/^^-^ mice, being in line with the initial clinical transplantation results (Garuti et al., 2011). Surprisingly, liver macrophages (Kupffer cells) were not rescued by the action of the donor, wild type hepatocytic-*Hfe* (Garuti et al., 2011).

These observations bring up a tantalizing thought whether Hfe is only required for the up regulation of hepcidin in response to elevated iron levels and thus for the maintenance of cellular and systemic iron pool, or the functions of this MHC-class I like molecule may be uncoupled from the control of iron homeostasis. Understanding distinct pathways that Hfe employs beyond the hepatocytes and beyond hepcidin regulation may leap translational medicine research in that some symptoms assigned to HH as a consequence of iron overload may be recognized appropriately and may better be treated by organ-specific therapies rather than systemic iron-depletion.

## Conflict of Interest Statement

The authors declare that the research was conducted in the absence of any commercial or financial relationships that could be construed as a potential conflict of interest.
